# Efficacy and safety of transarterial chemoembolization combined with donafenib and camrelizumab for hepatocellular carcinoma with portal vein tumor thrombus: a dual-center, retrospective propensity score-matched analysis

**DOI:** 10.3389/fimmu.2026.1882605

**Published:** 2026-08-05

**Authors:** Qi Zhang, Hezi Qing, Zhenwu Lei, Xingwu Xie

**Affiliations:** 1 Department of Interventional Radiology, Renmin Hospital, Hubei University of Medicine, Shiyan, Hubei, China; 2 Dongguan Community Health Service Center, Hanzhong, Shaanxi, China; 3 Department of Interventional Radiology, Qinghai University Affiliated Hospital, Xining, Qinghai, China

**Keywords:** camrelizumab, donafenib, hepatocellular carcinoma, portal vein tumor thrombus, propensity score matching

## Abstract

**Aim:**

To evaluate the efficacy and safety of transarterial chemoembolization combined with donafenib and camrelizumab (TACE+D+C) versus TACE plus donafenib (TACE+D) in the treatment of hepatocellular carcinoma (HCC) with portal vein tumor thrombus (PVTT).

**Methods:**

Patients with HCC complicated by PVTT diagnosed at two medical centers between December 2021 and July 2025 were enrolled and assigned to the TACE+D group and the TACE+D+C group. Propensity score matching (PSM) was performed at a 1:1 ratio with a caliper width of 0.2 standard deviations to balance baseline confounding factors, with 109 patients included in each group after matching. The primary endpoints were median overall survival (mOS) and median progression-free survival (mPFS), both calculated from the date of TACE completion. The secondary endpoints comprised objective response rate (ORR), disease control rate (DCR), and adverse events (AEs).

**Results:**

A total of 109 patients were enrolled in each group following PSM. In our retrospective PSM analysis, we observed that the TACE+D+C group was associated with longer mOS (15.90 vs 9.10 months, *P* < 0.001) and mPFS (11.20 vs 7.10 months, *P* < 0.001) relative to the TACE+D group. For intrahepatic lesions, the TACE+D+C group was associated with markedly higher ORR (58.72% vs 36.70%, *P* = 0.001) and DCR (89.91% vs 77.06%, *P* = 0.011). In terms of PVTT, the TACE+D+C group also presented higher ORR (42.20% vs 23.85%, *P* = 0.004) and DCR (77.06% vs 64.22%, *P* = 0.037). The incidence of AEs after TACE was similar in both groups; mild grade 1–2 immune-related adverse events (irAEs) unique to camrelizumab were only detected in the TACE+D+C group, with no severe grade 3–4 irAEs recorded.

**Conclusion:**

This retrospective PSM analysis observed a correlation between TACE+D+C triple therapy and longer survival and higher tumor response among PVTT-positive HCC patients. Given the inherent limitations of a retrospective observational design, these results cannot establish therapeutic superiority, and prospective randomized controlled trials are warranted to validate causal clinical benefits.

## Introduction

Hepatocellular carcinoma (HCC) is a leading global malignancy, ranking sixth in incidence and third in mortality worldwide, with a 5-year overall survival (OS) rate of only 18% (1, 2). Portal vein tumor thrombus (PVTT) is detected in 10%-40% of patients at the initial diagnosis of HCC, with 10%-15% of these patients presenting tumor invasion into the main portal vein trunk (3). Given the high prevalence of chronic hepatitis B virus (HBV) infection and suboptimal early screening practices in China, the proportion of PVTT among Chinese HCC patients is markedly higher, ranging from 44.0% to 62.2% (4). Cumulative clinical evidence indicates that PVTT obstructs portal venous perfusion, triggering intrahepatic congestion, portal hypertension, and subsequent liver dysfunction. In addition, PVTT facilitates intrahepatic lesion dissemination and distant metastasis, leaving patients with a median (mOS) of only 2–4 months under natural disease progression. Even with sorafenib, the conventional first-line tyrosine kinase inhibitor (TKI), the mOS of these patients is only prolonged to 5.6-8.1 months (5–7). These limited survival outcomes highlight a critical unmet clinical need for more effective therapeutic strategies for PVTT-positive HCC patients.

Transarterial chemoembolization (TACE) is a standard therapeutic option for intermediate and advanced HCC. However, PVTT-associated vascular invasion reshapes tumor vascularity and facilitates thrombus dissemination within the portal venous system, rendering single-agent TACE insufficient for complete tumor eradication and associated with suboptimal clinical responses (8). In recent years, advances in combination therapeutic strategies have offered new hope to resolve this clinical dilemma. Multiple prior clinical analyses have observed that TACE combined with targeted or immunotherapy shows correlatively longer survival endpoints compared with TACE monotherapy among patients with HCC (9, 10). It is well established in the literature that TACE-induced immunogenic cell death (ICD) can release tumor-associated antigens, converting the immune ‘cold tumor’ microenvironment into a ‘hot tumor’ phenotype. Additionally, this combination approach has been reported to reduce the proportion of immunosuppressive cells such as regulatory T cells (Tregs), thereby providing a favorable immune milieu to amplify the antitumor effects of targeted and immunotherapeutic agents (11). Based on this literature-derived rationale, the rational integration of TACE with systemic therapy, which is proposed to suppress the progression of primary tumors and PVTT through putative synergistic mechanisms, has emerged as a major research hotspot and promising developmental direction in the therapeutic management of PVTT-positive HCC patients.

Donafenib is a novel molecular targeted agent structurally optimized by deuterium substitution of the methoxy group in sorafenib, serving as a next-generation alternative to sorafenib for hepatocellular carcinoma treatment (12). The phase II-III real-world ZGDH3 trial demonstrated that donafenib was correlated with longer mOS relative to sorafenib. The 18-month survival rate was markedly higher in the donafenib group than in the sorafenib group (35.4% vs 28.1%, *P* = 0.046). Subgroup analyses of the same trial further suggested a consistent mOS advantage associated with donafenib (12.1 months vs 10.3 months, Hazard ratio (HR) = 0.831, *P* < 0.036). Notably, among patients with PVTT, accounting for 21.3% of the overall cohort, patients receiving donafenib showed a correlative trend of longer mOS. Relative to sorafenib (8.6 months vs 7.2 months), indicating its promising therapeutic potential in this specific patient subgroup (12). Camrelizumab was selected as the paired programmed death-1 (PD-1) inhibitor based on multiple evidence-based considerations. The combination of camrelizumab and an anti-angiogenic TKI has been approved by the National Medical Products Administration (NMPA) for first-line treatment of unresectable advanced HCC, supported by pivotal phase III registration trial data (13). Considering that over 80% of HCC cases in China are HBV-associated and most PVTT events occur in this subset, domestic real-world cohort studies have identified favorable outcomes with camrelizumab, generating substantial evidence specifically for this comorbid condition (14). Additionally, camrelizumab remodels the hypoxic microenvironment, modulates the proportions of immunosuppressive cells (e.g., M2 macrophages and Tregs), upregulates tumor programmed death-ligand 1 (PD-L1) expression, and facilitates infiltration and activation of immune effector cells, further reversing vascular endothelial growth factor (VEGF)-related immune suppression and potentiating the anti-angiogenic efficacy of donafenib (15). Cumulative clinical real-world data suggest that TACE combined with donafenib (TACE+D) and immune checkpoint inhibitors (ICIs) may correlate with longer OS and progression-free survival (PFS) among patients with initially unresectable HCC (16, 17).

Nevertheless, limited retrospective evidence is currently available regarding survival and tumor response in patients with HCC complicated by PVTT who received TACE+D+C. Furthermore, whether the TACE+D+C regimen confers a potential survival benefit over the standard TACE+D remains uncertain. In addition, the impact of clinical heterogeneity (including liver functional reserve and PVTT classification, such as Cheng’s classification (18) and Japanese Vein Portal (VP) classification (19)) on treatment response in this population remains undefined. Existing real-world reports of TACE-TKI-immune triple regimens have several notable limitations. Most prior cohorts primarily enrolled low-risk patients with preserved liver function, limited distant metastasis, and early-stage VP I-II PVTT, which does not mirror the advanced, high-tumor-burden population routinely encountered in clinical practice (20, 21). In addition, most triple-combination studies adopt lenvatinib as the anti-angiogenic backbone (22); real-world data of donafenib-based TACE+D+C for PVTT patients are scarce. Few prior studies have performed formal interaction analyses to screen subgroups with differential treatment responses (16). From a statistical perspective, most retrospective comparative studies only implemented basic propensity score matching (PSM) without supplementary robustness assessments, including inverse probability of treatment weighting (IPTW) and Rosenbaum sensitivity analysis, such that survival comparisons may be affected by unmeasured residual confounding (21, 23). To address the above limitations of previous studies, this dual-center retrospective PSM cohort employed a rigorous 1:1 matching design to explore associations between TACE+D+C triple therapy and clinical endpoints, and to systematically describe differences in survival, tumor response, and safety between the TACE+D and TACE+D+C groups among PVTT-positive HCC patients. Multi-layer sensitivity analyses and treatment interaction tests across key clinical stratifiers were performed. Tumor shrinkage of intrahepatic lesions and portal thrombus was assessed separately per modified Response Evaluation Criteria in Solid Tumors (mRECIST) (24) to provide thrombus-specific efficacy data.

In view of the above research gaps, this study adopted PSM to reduce confounding bias. We aimed to compare survival and tumor response outcomes associated with the TACE+D+C regimen versus the TACE+D regimen among HCC patients complicated by PVTT.

## Materials and methods

### Study population

A retrospective analysis was conducted to enroll patients with a confirmed diagnosis of HCC complicated by PVTT who received TACE treatment at the Department of Interventional Radiology of Shiyan People’s Hospital and the Affiliated Hospital of Qinghai University between December 2021 and July 2025. Baseline clinical data of all enrolled patients were systematically collected. Eligible patients were assigned to two groups: the TACE+D group and the TACE+D+C group. Regular follow-up was performed to collect serial imaging findings and laboratory examination results; treatment-related adverse events (TRAEs) were continuously documented. This study was approved by the Ethics Committee of the Affiliated Hospital of Qinghai University (Ethics Approval No.: P-SL-2023-421) and the Ethics Committee of Shiyan People’s Hospital (Ethics Approval No.: SYSRMYY-KYXS-2024-109).

HCC diagnosis was established based on dynamic contrast-enhanced CT, contrast-enhanced MRI, ultrasonography, and other imaging modalities. Patients were diagnosed if HCC characteristic manifestations were confirmed by at least two independent imaging examinations, accompanied by serum alpha-fetoprotein (AFP) level ≥ 400 ng/mL. For AFP-negative patients, an elevated prothrombin level served as an alternative diagnostic indicator; diagnosis could also be made when three imaging examinations showed typical HCC features. For patients who could not be definitively diagnosed via the above imaging and serological assessments, pathological biopsy was performed to confirm the final diagnosis (2).

### Inclusion criteria

1. Patients with a definitive diagnosis of HCC staged as China Liver Cancer Staging (CNLC) stage IIb or IIIa based on imaging examinations and laboratory tests; 2. Age ranging from over 18 years to under 75 years; 3. Absence of clear indications for surgical resection; 4. Preoperative liver function was classified as Child-Pugh grade A or B, with an Eastern Cooperative Oncology Group (ECOG) score of 0 to 1; 5. Patients with solitary or multiple HCC unsuitable for surgical resection or local ablation, and tumor burden occupying less than 50% of the total liver volume; 6. Presence of at least one measurable lesion defined by the mRECIST criteria (24) on CT or MRI; 7. No prior receipt of other anti-tumor related treatments.

### Exclusion criteria

1. Patients with metastatic liver disease originating from non-hepatic primary malignancies; 2. Individuals with Child-Pugh class C liver function; 3. Prior exposure to immunotherapeutic or targeted anticancer agents; 4. Clinically significant hepatic or renal dysfunction, or severe coagulopathy; 5. Patients who underwent surgical resection following initial combined-modality therapy. 6. For patients who experienced severe clinical deterioration or all-cause death following TACE and before their scheduled administration of donafenib or camrelizumab.

Treatment was discontinued if patients experienced grade > 4 adverse events (AEs) during the therapeutic course, which were assessed in accordance with the Common Terminology Criteria for Adverse Events Version 5.0 (CTCAE 5.0) (25). Additional trial termination criteria included voluntary withdrawal from treatment by the patient, occurrence of pregnancy among enrolled participants, overall deterioration of physical condition that precluded continued trial participation, and severe tumor progression accompanied by a marked decline in general health during medication. The final follow-up cutoff date was set as January 2026.

### PVTT was classified according to the Japanese vein portal classification system, with the following stratification

VP0, no PVTT; VP1, tumor thrombus confined to the third-order or more distal branches of the portal vein; VP2, tumor thrombus involving the second-order portal vein branches; VP3, tumor thrombus invading the first-order portal vein branches; VP4, tumor thrombus occupying the main portal vein trunk or the contralateral first-order portal vein branches. In this study, the VP 1 and VP 2 groups were categorized into the PVTT I-II group, while the VP 3 and VP 4 groups were categorized into the PVTT III-IV group.

This study uniformly adopted the VP classification proposed by the Liver Cancer Study Group of Japan (19) as the staging criterion for all analyses. This consistent classification standard was applied to all baseline comparisons, survival analyses, and Cox regression models, as well as all tables, figures, and **Supplementary Materials** throughout the manuscript.

### Treatment regimens

#### TACE protocol

All patients received TACE treatment, and the TACE procedures were performed by senior interventional physicians (**Supplementary Materials** for details).

#### Treatment regimen of donafenib combined with camrelizumab

Seven days after the completion of TACE, patients were administered oral donafenib (26) tosylate (brand name: Zepsun; Suzhou Zelgen Biopharmaceuticals Co., Ltd.) at a dose of 200 mg twice daily. We selected the 7-day interval between TACE and donafenib initiation based on safety and mechanistic evidence from domestic clinical guidelines and real-world cohorts. TACE-induced hepatic injury and post-embolization syndrome risk overlapping liver toxicity, with early anti-angiogenic therapy; Chinese TACE guidelines recommend a short recovery window, and a 7-day waiting period is widely adopted in donafenib combination studies (27). Mechanistically, TACE-triggered immunogenic cell death peaks at 5–7 days, and delayed donafenib administration relieves intratumoral hypoxia to amplify anti-tumor immunity (28). Multiple real-world and single-arm trials of triple therapy for PVTT-positive HCC adopt this timing strategy (10). For patients with intolerable AEs, the dose was reduced to 100 mg twice daily alongside standard symptomatic supportive care. Discontinuation of donafenib was mandated if tolerability remained poor following dose adjustment and symptomatic treatment.

Camrelizumab (29) 200 mg was administered via intravenous infusion 14 days after TACE.

This 200 mg once-every-21-day dosing schedule complies with the official prescribing information and published HCC combination trial protocols (29). Intravenous infusion requires close monitoring for hypersensitivity and immune-related AEs (irAEs); severe infusion reactions mandate permanent drug discontinuation per toxicology guidelines (30). Patients were closely monitored for hypersensitivity reactions, and treatment was continued every 21 days thereafter in the absence of abnormal reactions; otherwise, camrelizumab was permanently discontinued.

### Study endpoints

#### Primary endpoint

OS: defined as the time from the date of TACE completion to all-cause death.
PFS: defined as the interval from the date of TACE completion to the first documentation of objective tumor progression or all-cause death, whichever occurred first.

#### Secondary endpoints

AEs associated with TACE, targeted therapy, and immunotherapy (including nausea, vomiting, hand-foot skin reaction, etc.) were recorded and graded using the CTCAE 5.0 (25). Grade 1–2 AEs were managed with symptomatic treatment. For grade 3–4 AEs, the drug dose was first reduced by half, followed by symptomatic treatment.

### Assessment of therapeutic response

Tumor response to treatment was assessed based on mRECIST (24).

Standardized operational definitions of PVTT response per mRECIST.

Tumor and PVTT responses were strictly judged according to the mRECIST for HCC.

Complete response (CR): Disappearance of all arterial-enhancing intrahepatic lesions and complete resolution of arterial enhancement within PVTT;
Partial response (PR): ≥ 30% reduction in the sum of the longest diameters of viable arterial-enhancing lesions, accompanied by ≥ 30% shrinkage of enhanced PVTT;
Stable disease (SD): Insufficient shrinkage to meet PR criteria and no new lesions/PVTT progression;
Progressive disease (PD): ≥ 20% increase in viable lesion diameter, obvious enlargement of enhanced PVTT, or emergence of new intrahepatic/extrahepatic lesions.

#### Efficacy evaluation endpoints

The objective response rate (ORR) was calculated as: ORR = (CR+PR)/total enrolled cases×100%.
The disease control rate (DCR) was calculated as: DCR = (CR+PR+SD)/total enrolled cases×100%.

### Diagnostic criteria for immune-related pneumonitis

1. All patients with abnormal pulmonary imaging examinations received serum infectious marker tests, sputum bacterial and fungal culture, and respiratory virus nucleic acid detection to rule out bacterial, fungal, and viral pulmonary infections. 2. Chest contrast-enhanced CT scans revealed typical interstitial lesions characteristic of immune checkpoint inhibitor-related pneumonitis. 3. No pathogenic microorganisms were identified via laboratory tests. Clinical symptoms of patients were rapidly relieved after low-dose glucocorticoid monotherapy, without administration of anti-infective agents.

### Follow-up

Treatment response was assessed via triple-phase contrast-enhanced abdominal CT/MRI at 1-month post-TACE. All survival endpoints (OS and PFS) were calculated with the date of TACE completion as the unified time origin for both study groups, and regular follow-up surveillance was initiated on the same day. All imaging datasets were independently reviewed by two senior abdominal radiologists blinded to patient grouping and clinical outcomes, and tumor response was strictly judged per the mRECIST (24). Disagreements between the two readers were resolved via joint re-evaluation by a third chief radiologist with over 10 years of specialized experience in liver tumor imaging; the final consensus classification was adopted as the definitive objective response. If tumor progression occurred, TACE was administered at the patient’s discretion, and the patients continued to take donafenib and camrelizumab during this period. The time to disease progression and OS time were documented in detail. For patients without tumor progression, regular re-examinations were conducted every three months thereafter. The follow-up endpoint was set as January 2026.

### Statistical methods

Statistical analyses were performed using R (4.4.1). Categorical data were described as (n%), and the chi-square test or Fisher’s exact test was used for statistical comparison between the two groups. To minimize potential confounding bias between the two groups, PSM analysis was performed (23). In this study, 1:1 nearest-neighbor PSM was performed with a caliper of 0.2 standard deviations of the propensity score (**Supplementary Materials** for details). The Kaplan-Meier method and the log-rank test were applied to compare OS and PFS between the two groups. The Kaplan-Meier survival curves were plotted using the ggsurvplot package, and subgroup forest plots were generated via the ggplot2 package. In univariate Cox regression analysis, variables with *P* < 0.1 were included in the multivariate Cox regression model, followed by bidirectional stepwise regression. Schoenfeld residual tests were first conducted to verify the proportional hazards (PH) assumption before Cox regression modelling for OS and PFS. All covariates yielded *P* > 0.05, confirming the PH assumption held for both endpoints. This study adopted a 1:1 PSM design for comparative survival analysis. For all subsequent univariate and bidirectional stepwise multivariate Cox regression models, cluster-adjusted robust sandwich standard errors clustered by matched pairs were applied to address the non-independence of paired samples from matching. Conventional unadjusted standard errors were also presented alongside robust outputs for comparative reference. Covariates with a two-sided univariate *P* < 0.1 were eligible for entry into the multivariate stepwise models. A two-sided *P* < 0.05 was defined as the threshold of statistical significance.

## Results

### Baseline characteristics of patients

**Figure 1:**
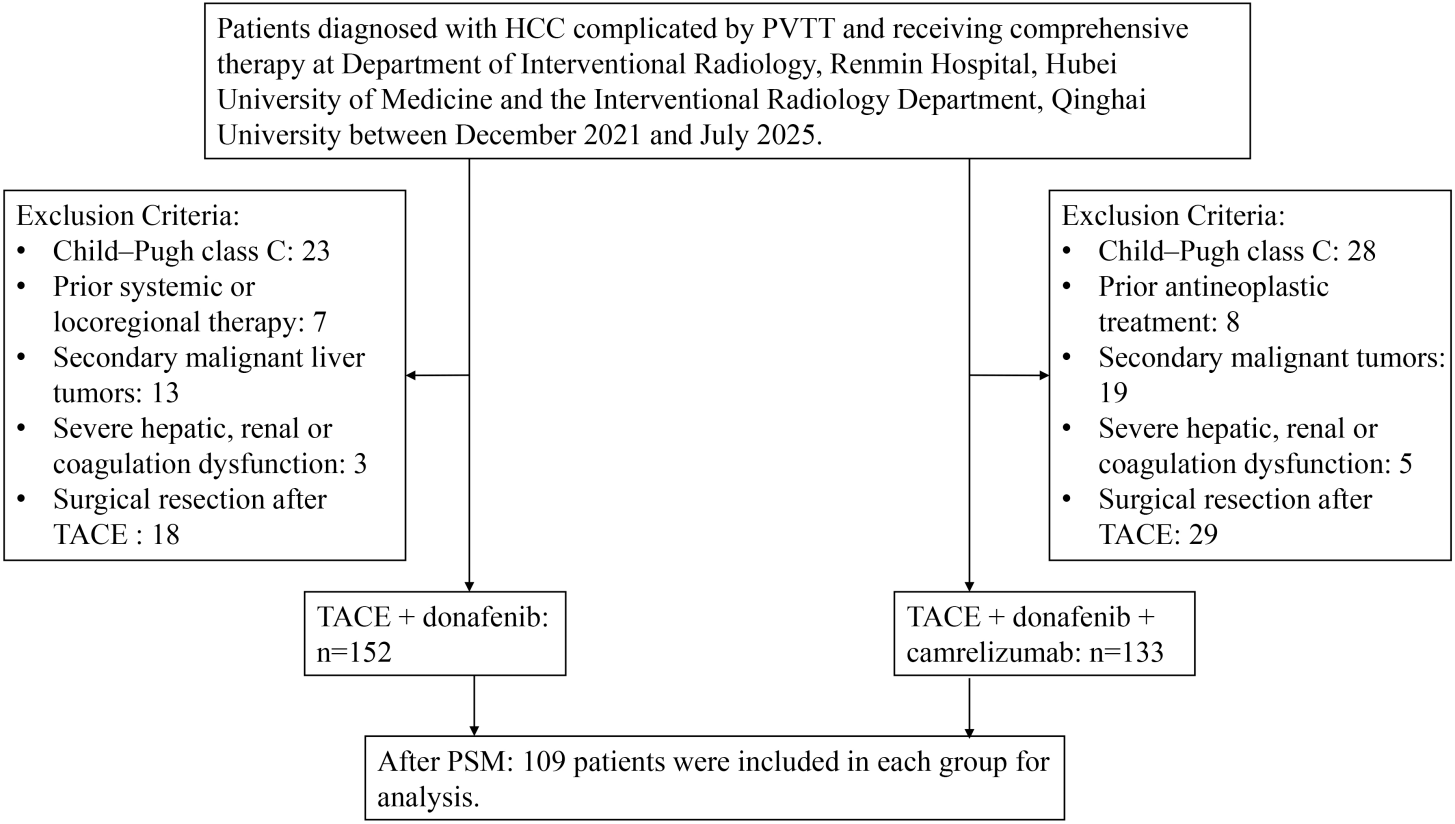
Patient enrollment flowchart. HCC, Hepatocellular Carcinoma; PVTT, Portal Vein Tumor Thrombus; TACE, Transarterial Chemoembolization; PSM, Propensity Score Matching.

Following rigorous enrollment based on established inclusion and exclusion criteria, 152 patients were allocated to the TACE+D cohort and 133 patients to the TACE+D+C cohort (**Figure 1**). Before PSM, statistically significant discrepancies in AFP and PVTT were identified between the two groups, while the remaining baseline indicators were comparable. After PSM adjustment, 109 participants were successfully matched in each cohort, and all baseline covariates achieved satisfactory intergroup balance with no statistically significant differences (**Table 1**).

**Table 1:** Baseline clinical characteristics of enrolled patients.

Variables	Before PSM		*P*	SMD	After PSM		*P*	SMD
	TACE+D, (n=152), n, (%)	TACE+D+C (n=133), n, (%)			TACE+D, (n=109), n, (%)	TACE+D+C (n=109), n, (%)		
Age (years)			0.098	0.125			0.653	0.059
≤ 40	25 (16.45)	13 (9.77)			10 (9.17)	12 (11.01)		
> 40	127 (83.55)	120 (90.23)			99 (90.83)	97 (88.99)		
Sex			0.232	0.150			0.744	0.045
Female	40 (26.32)	27 (20.30)			25 (22.94)	23 (21.10)		
Male	112 (73.68)	106 (79.70)			84 (77.06)	86 (78.90)		
Child-Pugh class			0.705	0.045			0.887	0.049
A	57 (37.50)	47 (35.34)			38 (34.86)	39 (35.78)		
B	95 (62.50)	86 (64.66)			71 (65.14)	70 (64.22)		
HBV			0.081	0.085			0.747	0.043
No	29 (19.08)	37 (27.82)			24 (22.02)	26 (23.85)		
Yes	123 (80.92)	96 (72.18)			85 (77.98)	83 (76.15)		
AFP (ng/mL)			0.006	0.148			0.775	0.039
≤ 400	70 (46.05)	40 (30.08)			36 (33.03)	38 (34.86)		
> 400	82 (53.95)	93 (69.92)			73 (66.97)	71 (65.14)		
ALT (U/L)			0.285	0.123			0.751	0.043
≤ 35	35 (23.03)	38 (28.57)			25 (22.94)	27 (24.77)		
> 35	117 (76.97)	95 (71.43)			84 (77.06)	82 (75.23)		
ECOG score			0.825	0.026			0.873	0.022
0	120 (78.95)	103 (77.44)			83 (76.15)	84 (77.06)		
1	32 (21.05)	30 (22.56)			26 (23.85)	25 (22.94)		
Albumin (g/L)			0.759	0.049			0.683	0.055
≤ 30	74 (48.68)	52 (39.10)			51 (46.79)	48 (44.04)		
> 30	78 (51.32)	81 (60.90)			58 (53.21)	61 (55.96)		
Extrahepatic metastasis			0.752	0.037			0.766	0.040
Yes	110 (72.37)	94 (70.68)			78 (71.56)	76 (69.72)		
No	42 (27.63)	39 (29.32)			31 (28.44)	33 (30.28)		
Number of tumors			0.173	0.12			0.879	0.021
≤ 3	115 (75.66)	91 (68.94)			80 (73.39)	79 (72.48)		
> 3	37 (24.34)	42 (31.56)			29 (26.61)	30 (27.52)		
Tumor size			0.117	0.128			0.175	0.091
≤ 7cm	64 (42.11)	44 (33.08)			26 (23.85)	35 (32.11)		
> 7cm	88 (57.89)	89 (66.92)			83 (76.15)	74 (67.89)		
PVTT			0.040	0.142			0.327	0.074
I-II	86 (56.58)	59 (44.36)			37 (33.94)	44 (40.37)		
III-IV	66 (43.42)	74 (55.64)			72 (66.06)	65 (59.63)		

HBV, Hepatitis B Virus; ECOG, Eastern Cooperative Oncology Group; PVTT, Portal Vein Tumor Thrombus; PSM, Propensity Score Matching; AFP, Alpha-Fetoprotein; ALT, Alanine Aminotransferase; SMD, standardized mean difference; TACE+D, transarterial chemoembolization plus donafenib; TACE+D+C, transarterial chemoembolization combined with donafenib and camrelizumab.

### Survival outcomes

The median follow-up duration was 13.92 months (range, 4.64-23.17 months) in the TACE+D+C group, and 10.43 months (range, 4.41-18.45 months) in the TACE+D group.

**Figure 2:**
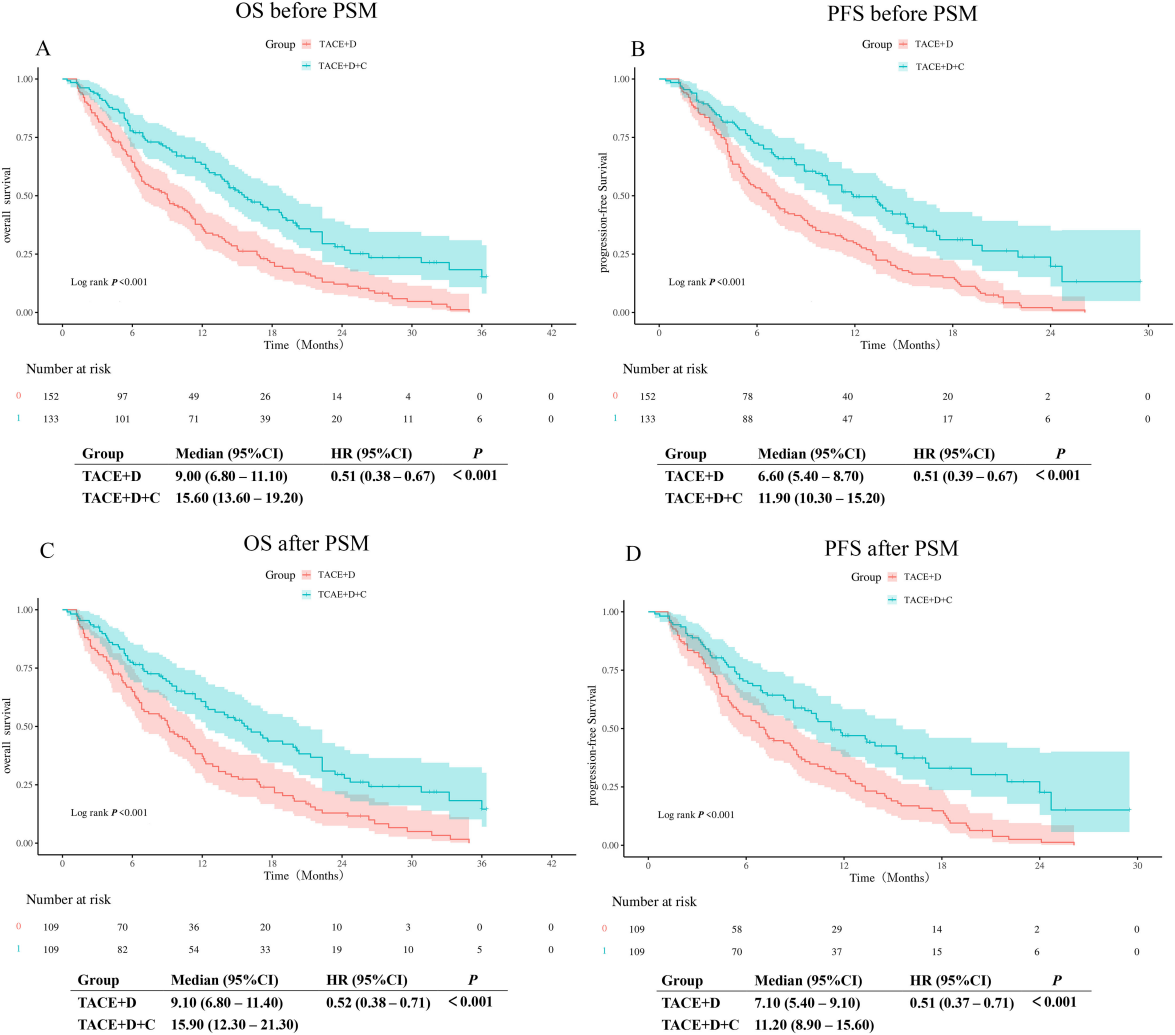
Kaplan-Meier survival curves of OS and PFS between the TACE+D and TACE+D+C groups before and after PSM. **(A)** OS before PSM; **(B)** PFS before PSM; **(C)** OS after PSM; **(D)** PFS after PSM. TACE+D, transarterial chemoembolization plus donafenib; TACE+D+C, transarterial chemoembolization combined with donafenib and camrelizumab; HR, hazard ratio; CI, confidence interval; PSM, propensity score matching.

Before PSM, the mOS was significantly longer in the TACE+D+C group than in the TACE+D group (15.60 months, 95% confidence intervals (CI): 13.60-19.20 vs 9.00 months, 95% CI: 6.80-11.10; HR = 0.51, 95% CI: 0.38-0.67, *P* < 0.001) (**Figure 2A**). Similarly, the median PFS (mPFS) was markedly prolonged in the TACE+D+C group relative to the TACE+D group (11.90 months, 95% CI: 10.30-15.20 vs 6.60 months, 95% CI: 5.40-8.70; HR = 0.51, 95% CI: 0.39-0.67, *P* < 0.001) (**Figure 2B**).

After PSM adjustment, the TACE+D+C group still presented a substantially superior mOS compared with the TACE+D group (15.90 months, 95% CI: 12.30-21.30 vs 9.10 months, 95% CI: 6.80-11.40; HR = 0.52, 95% CI: 0.38-0.71, *P* < 0.001) (**Figure 2C**). Consistently, mPFS remained significantly prolonged in the TACE+D+C group (11.20 months, 95% CI: 8.90-15.60) versus the TACE+D group (7.10 months, 95% CI: 5.40-9.10; HR = 0.51, 95% CI: 0.37-0.71, *P* < 0.001) (**Figure 2D**).

### Events and censoring distribution for OS and PFS after PSM

After 1:1PSM, 109 patients were included in each group. Median follow-up time was calculated using reverse Kaplan-Meier estimation. For OS, the TACE+D group had 95 death events and 14 censored cases (censoring rate 12.84%, median censoring time 10.5 months), whereas the TACE+D+C group had 70 death events and 39 censored cases (censoring rate 35.78%, median censoring time 15.2 months). For PFS, there were 101 progression events and 8 censored cases (censoring rate 7.34%, median censoring time 7.55 months) in the TACE+D group, and 64 progression events and 45 censored cases (censoring rate 41.28%, median censoring time 12.30 months) in the TACE+D+C group. All censoring events were attributed to incomplete follow-up before the January 2026 data cut-off, with no loss to follow-up (**Supplementary Materials**: Events and censoring distribution for OS and PFS after PSM).

### Subgroup analysis

In the overall cohort (n = 218), receipt of the TACE+D+C regimen was associated with longer OS and a 48% lower relative risk of all-cause death (HR = 0.52, 95% CI: 0.38-0.71, *P* = 0.001). Of note, all subgroup stratification analyses and interaction tests were post-hoc exploratory analyses, with no prespecified statistical hypotheses and no adjustment for multiple comparisons. Potential heterogeneous survival trends were noted across different patient populations in subgroup analyses. Significant interaction effects between treatment efficacy and ECOG score, tumor size, and PVTT were observed, with interaction *P* values of 0.049, 0.033, and 0.006, respectively. Patients with an ECOG score of 0 showed a correlative trend of longer survival when receiving triple therapy (HR = 0.45, 95% CI: 0.31-0.65, *P* = 0.001), whereas no meaningful therapeutic disparity was detected in patients with an ECOG score of 1 (HR = 0.90, 95% CI: 0.48-1.71, *P* = 0.754). Numerically longer survival was noted in patients with tumor size > 7 cm (HR = 0.42, 95% CI: 0.28-0.61, *P* = 0.001), while no statistically significant survival difference existed for patients with tumor size ≤ 7 cm (HR = 0.84, 95% CI: 0.47-1.49, *P* = 0.556). PVTT grade III-IV patients exhibited correlatively prolonged survival linked to triple therapy (HR = 0.37, 95% CI: 0.24-0.55, *P* = 0.001), yet no significant intergroup survival gap was identified in patients with low-grade PVTT (I-II; HR = 0.79, 95% CI: 0.48-1.31, *P* = 0.363) (**Supplementary Figure 1**).

### Stratified analysis

In the subgroup of patients with an ECOG performance status of 0, the TACE+D+C group was associated with longer mOS (18.90 months vs 9.60 months, log-rank *P* < 0.001) and mPFS (15.20 months vs 7.30 months, log-rank *P* < 0.001) relative to the TACE+D group (**Supplementary Figures 2A, E**). In contrast, among patients with an ECOG performance status of 1, no significant differences were observed between the TACE+D+C and TACE+D groups in terms of mOS (7.30 months vs 6.30 months, log-rank *P* = 0.750) and mPFS (5.60 months vs 4.40 months, log-rank *P* = 0.920) (**Supplementary Figures 2B, F**).

In the subgroup with tumor diameter ≤ 7 cm, no statistically significant differences were detected between the TACE+D+C and TACE+D groups in terms of mOS (17.20 months vs 18.20 months, log-rank *P* = 0.556) and mPFS (13.90 months vs 13.30 months, log-rank *P* = 0.503) (**Supplementary Figures 2C, G**). For patients with tumor diameter >7 cm, the TACE+D+C group was associated with markedly prolonged mOS (14.20 months vs 6.80 months, log-rank *P* < 0.001) and mPFS (10.40 months vs 5.50 months, log-rank *P* < 0.001) compared with the TACE+D counterpart (**Supplementary Figures 2D, H**).

In the subgroup with PVTT grade I-II, mOS (20.30 months vs 18.90 months, log-rank *P* = 0.363) and mPFS (15.60 months vs 13.30 months, log-rank *P* = 0.078) were comparable between the TACE+D+C group and the TACE+D group (**Supplementary Figures 2I, K**). For patients presenting with PVTT grade III-IV, substantially longer mOS (11.90 months vs 6.00 months, log-rank *P* < 0.001) and mPFS (10.00 months vs 4.90 months, log-rank *P* < 0.001) were noted among the TACE+D+C cohort relative to the TACE+D group (**Supplementary Figures 2J, L**).

### Assessment of tumor response

**Figure 3:**
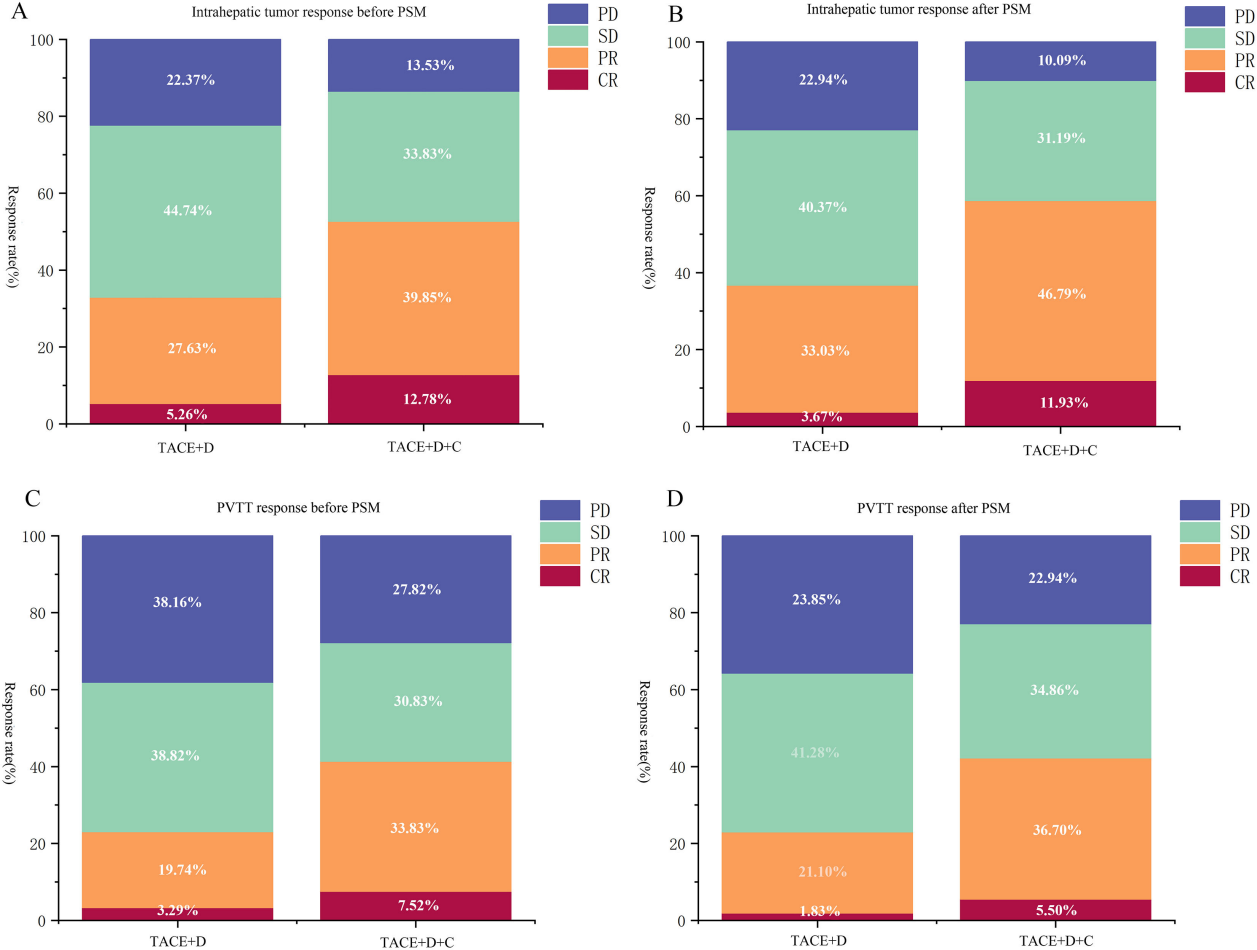
Tumor responses of intrahepatic tumor and PVTT between the TACE+D and the TACE+D+C groups before and after PSM. **(A)** Intrahepatic tumor before PSM; **(B)** intrahepatic tumor after PSM; **(C)** PVTT response before PSM; **(D)** PVTT response after PSM. TACE+D, transarterial chemoembolization plus donafenib; TACE+D+C, transarterial chemoembolization combined with donafenib and camrelizumab; PSM, propensity score matching; CR, complete response; PR, partial response; SD, stable disease; PD, progressive disease; ORR, objective response rate; DCR, disease control rate.

Tumor responses of intrahepatic lesions and PVTT were evaluated according to the mRECIST (**Figure 3**). Before PSM, statistically significant differences were observed between the TACE+D+C and TACE+D groups in terms of CR (12.78% vs 5.26%, *P* = 0.025), PR (39.85% vs 27.63%, *P* = 0.029), and ORR (52.63% vs 32.89%, *P* < 0.001). By contrast, no significant intergroup differences were found in PD (13.53% vs 22.37%, *P* = 0.054), SD (33.83% vs 44.74%, *P* = 0.061), and DCR (86.47% vs 77.63%, *P* = 0.054) (**Figure 3A**; **Supplementary Table 1**). Following PSM, the two groups exhibited significant discrepancies in CR (11.93% vs 3.67%, *P* = 0.023), PR (46.79% vs 33.03%, *P* = 0.038), PD (10.09% vs 22.94%, *P* = 0.011), ORR (58.72% vs 36.70%, *P* = 0.001), and DCR (89.91% vs 77.06%, *P* = 0.011). No statistical difference was detected in SD (31.19% vs 40.37%, *P* = 0.158) (**Figure 3B**; **Supplementary Table 1**).

Concerning PVTT evaluation before PSM, statistically significant differences between the TACE+D+C and TACE+D groups were noted in PR (33.83% vs 19.74%, *P* = 0.007) and ORR (41.35% vs 23.03%, *P* < 0.001). In contrast, no significant intergroup disparities were found in CR (7.52% vs 3.29%, *P* = 0.111), SD (30.83% vs 38.82%, *P* = 0.159), PD (27.82% vs 38.16%, *P* = 0.065), and DCR (72.18% vs 61.84%, *P* = 0.065) (**Figure 3C**; **Supplementary Table 2**). Following PSM, assessment of PVTT tumor response revealed significant differences between the two treatment arms in PR (36.70% vs 21.10%, *P* = 0.011), PD (22.94% vs 35.78%, *P* = 0.037), ORR (42.20% vs 23.85%, *P* = 0.004), and DCR (77.06% vs 64.22%, *P* = 0.037). No statistically meaningful divergence was observed for CR (5.50% vs 1.83%, *P* = 0.280) and SD (34.86% vs 41.28%, *P* = 0.329) (**Figure 3D**; **Supplementary Table 2**).

### TRAEs

In the TACE+D+C group, the most common grade 1–2 TRAEs were hair loss (23.85%), diarrhea (14.68%), and hand-foot skin reaction (25.69%), while grade 3–4 TRAEs were predominantly diarrhea (2.75%) and hand-foot skin reaction (8.26%). IrAEs such as IRP, hypothyroidism, and reactive cutaneous capillary endothelial proliferation (RCCEP) occurred exclusively in the TACE+D+C triple regimen group. These AEs are well-documented, predictable on-target effects of camrelizumab. All irAEs were grade 1–2 and resolved with symptomatic treatment, with no grade 3–4 irAEs. In the TACE+D group, the most common grade 1–2 TRAEs were hair loss (22.02%), diarrhea (15.61%), and hand-foot skin reaction (24.77%), while grade 3–4 TRAEs were predominantly diarrhea (4.59%) and hand-foot skin reaction (7.34%). All grade 1–2 AEs were alleviated with symptomatic treatment, and grade 3–4 AEs improved after dose reduction combined with supportive care. In the TACE+D+C group, 20 patients (18.35%) showed alleviation of AEs following dose reduction and symptomatic treatment, while in the TACE+D group, 22 patients (20.18%) experienced similar improvement (**Supplementary Figure 3**; **Supplementary Table 3**).

### Schoenfeld residual tests

Schoenfeld residual tests were performed to verify the PH assumption for all Cox regression models. For both OS and PFS multivariable models, the correlation between Schoenfeld residuals and survival time was non-significant (all *P* > 0.05), confirming that the PH assumption was fully satisfied (Schoenfeld residual tests section in the **Supplementary Materials**).

### Cluster-Robust Cox Regression

**Figure 4:**
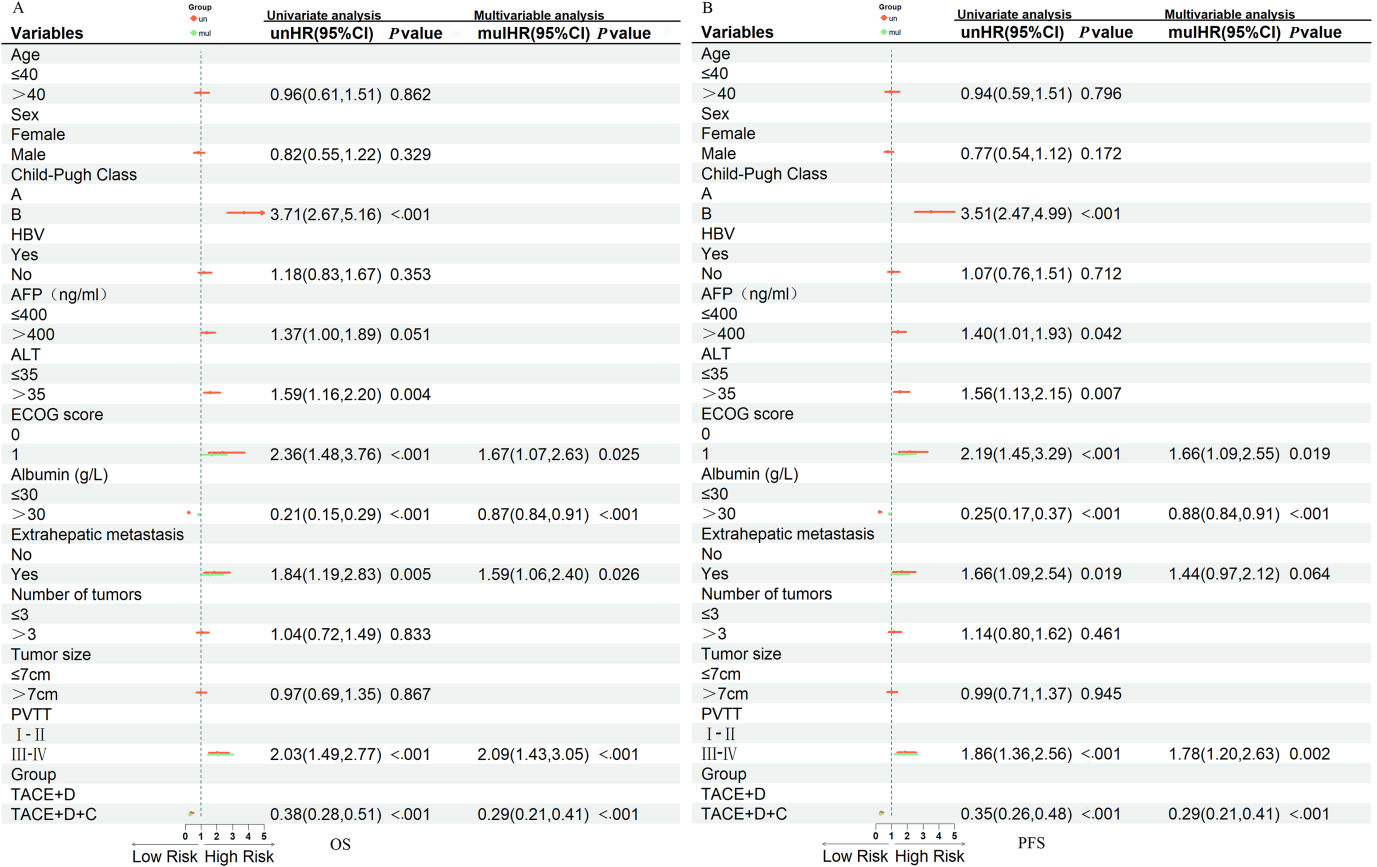
Cluster-robust univariate and multivariate cox regression analyses for OS **(A)** and PFS **(B)** after PSM. Reference groups for each categorical variable are defined as follows: ECOG score = 0; albumin ≤ 30 g/L; no extrahepatic metastasis; PVTT I-II; treatment group = TACE+D; Child-Pugh class A; AFP ≤ 400 ng/mL; ALT ≤ 35 U/L; age ≤ 40 years; number of tumors ≤ 3; tumor size ≤ 7 cm; sex = female. HR > 1 indicates a higher risk of endpoint events in the comparison group relative to the reference group; HR < 1 indicates a lower risk. Un, Univariate; Mul, Multivariate; TACE+D, transarterial chemoembolization plus donafenib; TACE+D+C, transarterial chemoembolization combined with donafenib and camrelizumab; PSM, Propensity Score Matching; PVTT, Portal Vein Tumor Thrombus; AFP, Alpha-Fetoprotein; ALT, Alanine Aminotransferase; OS, Overall Survival; PFS, Progression-Free Survival; HR, Hazard ratio.

After PSM, univariate and multivariate prognostic analyses were performed to identify predictors of OS and PFS, with the results presented in **Figure 4**. For the OS endpoint, ECOG score (1 versus reference 0; HR = 1.67, 95% CI: 1.07-2.63, *P* = 0.025), albumin (> 30 g/L versus reference ≤ 30 g/L; HR = 0.87, 95% CI: 0.84-0.91, *P* < 0.001), extrahepatic metastasis (yes versus reference no; HR = 1.59, 95% CI: 1.06-2.40, *P* = 0.026), PVTT (III-IV versus reference I-II; HR = 2.09, 95% CI: 1.43-3.05, *P* < 0.001), and treatment group (TACE+D+C versus reference TACE+D; HR = 0.29, 95% CI: 0.21-0.41, *P* < 0.001) were identified as independent prognostic factors for OS. (**Figure 4A**; **Supplementary Table 4**). Likewise, ECOG score (1 versus reference 0; HR = 1.66, 95% CI: 1.09-2.55, *P* = 0.019), albumin (> 30 g/L versus reference ≤ 30 g/L; HR = 0.88, 95% CI: 0.84-0.91, *P* < 0.001), extrahepatic metastasis (yes versus reference no; HR = 1.44, 95% CI: 0.97-2.12, *P* = 0.064), PVTT (III-IV versus reference I-II; HR = 1.78, 95% CI: 1.20-2.63, *P* = 0.002) and treatment group (TACE+D+C versus reference TACE+D; HR = 0.29, 95% CI: 0.21-0.41, *P* < 0.001) were identified as independent prognostic determinants for PFS (**Figure 4B**; **Supplementary Table 5**).

## Discussion

After balancing baseline confounding factors via PSM, this retrospective cohort study observed a correlative association with longer survival and numerically improved DCR in patients receiving TACE+D+C compared with those receiving TACE+D. Specifically, longer mOS (15.90 vs 9.10 months) and mPFS (11.20 vs 7.10 months), as well as higher ORR and DCR for both intrahepatic lesions and PVTT, were observed in the TACE+D+C group. The incidence and severity of AEs after TACE were comparable between the two cohorts. Mild grade 1–2 irAEs specific to camrelizumab occurred only in patients receiving the triple combination regimen. Notably, in the 1:2 PSM analysis, the improvement in mPFS with the TACE+D+C group did not reach statistical significance, likely due to residual baseline imbalances and reduced statistical power associated with the wider matching ratio. Nevertheless, in this retrospective cohort study, the 1:1 primary analysis, IPTW, and 1:1 matching analysis with a tighter caliper all consistently suggested that the TACE+D+C group might confer a PFS benefit over the TACE+D group. Collectively, these correlative findings are consistent with the robustness of our primary survival observations and suggest a potential association between the TACE+D+C group and improved efficacy relative to the TACE+D group in this high-risk cohort.

Although previous studies have preliminarily explored the clinical application of TACE combined with systemic therapy in HCC patients complicated with PVTT, most of these investigations enrolled predominantly low-risk populations, leaving the overall level of clinical evidence and general applicability to be further improved. Multicenter large-cohort studies conducted by Ran You et al. (20) and Shu-Qun Li et al. (21) have demonstrated that TACE combined with targeted immunotherapy confers significant improvements in OS and PFS compared with systemic therapy alone. Nevertheless, these studies are hampered by substantial enrollment selection bias and present notable inherent limitations. The cohort enrolled in the study by Ran You et al. (20) consisted predominantly of patients with Child-Pugh class A liver function (accounting for 76%-85%) and those with AFP levels ≤ 400 ng/mL (47%-50%). Similarly, the research reported by Shu-Qun Li et al. (21) recruited a high proportion of patients with favourable prognostic profiles, in which 78% presented with Child-Pugh class A and up to 92.7% had no extrahepatic metastasis. Such enrollment composition substantially deviates from the severe clinical characteristics of real-world HCC patients complicated with PVTT. In contrast, the present study specifically focused on high-risk real-world populations with unmet clinical needs. Only 35%-37% of enrolled patients were classified as Child-Pugh class A, and 70%-72% were complicated by extrahepatic metastasis. By integrating PSM, IPTW, and Rosenbaum sensitivity analysis, we established a rigorous statistical evidence framework that substantially mitigated selection and confounding biases in retrospective research. This retrospective study observed consistent correlative survival trends favoring the TACE+D+C group over the TACE+D group, with numerically improved intrahepatic ORR and mPFS when indirectly compared against the sorafenib plus PD-1 inhibitor plus TACE regimen described by Zou et al. (22). It should be noted that direct cross-study comparisons should be interpreted with caution, given the substantial heterogeneity across studies in enrollment criteria, baseline characteristics, and sample sizes. Nevertheless, a rough comparison with previously reported combination strategies may help contextualize the clinical value of the present regimen. Zhang et al. (31) reported a mPFS of 10.4 months and a mOS of 13.8 months with the sorafenib+TACE+Stereotactic Body Radiation Therapy (SBRT) regimen in PVTT-HCC patients; Huang et al. (32) observed a mPFS of 15.7 months and a 1-year OS rate of 83.3% with sorafenib+camrelizumab+TACE+SBRT in a small case series. For the lenvatinib plus TACE and PD-1 inhibitor regimen, the mPFS was 9.3 months, OS was 24.4 months, and ORR was 50.7% (33). In this retrospective analysis, our TACE+D+C group showed numerically higher mPFS (11.2 months) and intrahepatic ORR (58.72%), with a mOS (15.9 months) comparable to most reported outcomes. Furthermore, multiple donafenib-based combination studies have suggested antitumor activity of this agent across different local treatment modalities (34). Collectively, these cross-study comparisons may suggest potential clinical utility of the TACE+D+C triple regimen for patients with PVTT-HCC, although head-to-head prospective studies remain necessary to define its relative superiority.

In this retrospective cohort, the TACE+D+C regimen was associated with longer survival and improved tumor responses relative to the TACE+D regimen, a correlative trend directionally consistent with findings from previous studies. Prior studies have suggested that the combination of targeted therapy and immunotherapy may exert synergistic antiangiogenic effects, and this proposed synergy is largely attributed to the distinct yet complementary mechanisms of the two agents: donafenib provides sustained inhibition of VEGF, whereas camrelizumab activates T-cell-mediated antitumor immunity (29, 35). Donafenib, a deuterated sorafenib analogue with optimized pharmacokinetic properties, has been validated to inhibit VEGF signaling in preclinical studies (12). Notably, PVTT is characterized by excessive VEGF expression and severe intralesional hypoxia, two key pathological features that promote the accumulation of immunosuppressive cells and hinder the infiltration of effector T cells (36). Further preclinical evidence has demonstrated that sustained VEGF inhibition can normalize aberrant tumor vasculature, alleviate hypoxia within tumor thrombi, and reduce the infiltration of M2 macrophages and myeloid-derived suppressor cells (MDSCs), thereby relieving local immunosuppression in the PVTT microenvironment (37). Camrelizumab targets the PD-1/PD-L1 axis to reverse tumor immune escape (30). Existing studies have confirmed that PVTT tissues are enriched with C5aR^+^ M2 macrophages and present upregulated PD-L1 expression; this unique immune phenotype substantially suppresses the secretion of cytotoxic granzyme by CD8^+^ T cells and impairs antitumor immune responses (36). Preclinical models have further verified that PD-1 pathway blockade can restore the cytotoxic function of exhausted T cells (38). Accordingly, it is biologically plausible, based on preclinical evidence, that the combination of donafenib and camrelizumab may produce a hypothesized synergistic effect: donafenib-mediated vascular normalization might mitigate hypoxia-induced immunosuppression and facilitate effector T-cell infiltration, whereas camrelizumab might reverse T-cell exhaustion to activate antitumor immunity. However, as no direct mechanistic assays were performed in this retrospective study, this interpretation remains speculative and is presented only as a literature-based hypothesis to contextualize our correlative findings. Drawing on preclinical data, this putative synergistic mechanism offers a plausible biological hypothesis for the favorable tumor control outcomes observed in our cohort. However, without direct mechanistic or immune correlative evidence from the current dataset, this interpretation remains speculative and should be viewed only as a literature-based theoretical framework to contextualize our retrospective findings. PVTT is a common and critical complication of HCC, which typically signifies disease progression, limited treatment options, and a poor long-term prognosis (39). Currently, the clinical guidelines issued by the European Association for the Study of the Liver (EASL) (40), the American Association for the Study of Liver Diseases (AASLD) (41), and the Barcelona Clinic Liver Cancer (BCLC) staging system categorize PVTT as an absolute or relative contraindication to surgical resection and conventional TACE treatment. Mechanistically, PVTT disrupts hepatic hemodynamics, accelerates tumor dissemination, and induces immunosuppression by remodeling the immune microenvironment. These processes impair host anti-tumor immune responses and directly precipitate substantial deterioration of liver function (42, 43). Nevertheless, limited previous research has centered on immune-associated survival differences in patients with PVTT, which motivates the current analysis.

Exploratory subgroup analyses revealed heterogeneous correlative survival trends associated with receipt of TACE+D+C therapy among patients with HCC. Stratified outcomes showed that survival differences were particularly pronounced in patients with ECOG performance status of 0, tumor diameter > 7 cm, and grade III-IV PVTT (44, 45). Notably, these subgroup analyses were exploratory and not predefined in the original statistical plan; therefore, the observed survival patterns should not be overinterpreted or used to establish definitive patient selection criteria. Variations in treatment responses across different subgroups are likely driven by a combination of multiple clinical and biological factors. ECOG score is an important indicator for evaluating patients’ physical condition and hepatic functional reserve. Patients with ECOG score 0 generally have better organ function and immune status, and previous studies have demonstrated that such patients achieve superior OS and response rates compared with those with ECOG score 1 when treated with immune checkpoint inhibitors (46). In this retrospective study, subgroup analysis of the TACE+D+C treatment cohort showed that patients with ECOG score 0 derived significantly greater clinical benefit than those with ECOG score 1, which is consistent with the above evidence. This association may be partly explained by their more favorable immune status; however, this hypothesis requires prospective validation and external validation in an independent cohort. In terms of tumor burden and PVTT severity, larger tumor lesions and advanced-grade PVTT are closely correlated with a higher risk of microvascular invasion (MVI). Increased tumor diameter has been identified as an independent risk factor for MVI progression in HCC (47). Translational studies have further shown that large HCC lesions exhibit upregulated expression of VEGF and matrix metalloproteinases (MMPs); these molecular alterations promote tumor angiogenesis and extracellular matrix degradation, endowing tumors with stronger invasion and metastasis potential. This preclinical evidence may offer a literature-based speculative context for the correlative survival trends observed in our high tumor burden and advanced PVTT subgroups, but this speculation cannot be verified by the current observational data and requires future mechanistic investigations (48). In summary, the unique clinical and molecular characteristics of HCC patients with large tumors and advanced PVTT increase their susceptibility to MVI, tumor invasion, and distant metastasis, which may offer a clinical rationale for the correlative therapeutic trends observed in these specific subgroups, although this interpretation remains hypothetical and requires prospective mechanistic validation.

In terms of safety profile, hand-foot skin reaction (25.69%), diarrhea (14.68%), and hair loss (23.85%) were the most frequent AEs in the TACE+D+C group, and most AEs were grade 1-2. The dominant grade 3–4 AEs were diarrhea (2.75%) and hand-foot skin reaction (8.26%), and these symptoms improved after routine symptomatic care. A similar proportion of patients in both groups required dose reduction and supportive treatment for TRAEs: 18.35% in the TACE+D+C group and 20.18% in the TACE+D group experienced AEs relief after intervention. In the two cohorts, the incidence of moderate to severe AEs after TACE was similar, which is consistent with the published data on targeted combination therapy (12, 16). However, grade 1–2 irAEs unique to camrelizumab were only observed among patients receiving triple therapy. These irAEs are definite TRAEs stemming from camrelizumab blockade; all irAEs identified in this cohort were mild and resolved completely with standard management, with no high-grade immune complications observed. Taken together, TACE+D+C delivers acceptable clinical tolerability for PVTT-positive HCC patients, with comparable non-irAEs versus TACE+D and only manageable low-grade irAEs specific to camrelizumab added in the triple regimen.

This dual-center retrospective real-world cohort analysis provides efficacy and safety evidence for the triple regimen of TACE+D+C in patients with advanced HCC complicated by PVTT. Two external contextual factors should be taken into account when extrapolating the observational findings of this retrospective real-world study to other clinical settings. On the one hand, our cohort predominantly consisted of patients with HBV-related HCC, the dominant etiology of HCC across Asia, whereas HCC in Western populations is mostly attributable to metabolic or alcoholic liver disease (14). Etiologic heterogeneity may subtly alter tumor biological phenotypes and treatment tolerability, yet this triple combination remains a viable therapeutic option for non-HBV PVTT-positive HCC patients (36). On the other hand, donafenib and camrelizumab are domestically innovative agents with wide clinical availability and full medical insurance coverage in China (12, 33). However, divergent pharmaceutical policies worldwide lead to substantial disparities in their regulatory approval status and clinical accessibility across regions. Large-scale international multicenter retrospective or prospective trials enrolling PVTT-HCC patients with heterogeneous underlying liver diseases are warranted to further validate the generalizability of our real-world observations.

Although the present study provides valuable clinical evidence describing clinical outcomes in patients with HCC and PVTT, several inherent limitations should be acknowledged. First, this study is a retrospective observational investigation. Although 1:1 PSM was applied to balance baseline clinical characteristics between groups, matching cannot eliminate treatment selection bias, and unmeasured confounders may still confound our results. We therefore performed a Rosenbaum sensitivity analysis to quantify the magnitude of hidden confounding that would negate the significant intergroup survival differences, thereby assessing the robustness of our comparative outcomes. While PSM combined with Rosenbaum sensitivity analysis substantially mitigated biases from measured and potential unmeasured covariates, the retrospective design prevents us from establishing a causal link between triple combination therapy and improved survival. The observed intergroup disparities only demonstrate correlative associations, underscoring the necessity of prospective randomized controlled trials (RCTs) to verify the causal therapeutic benefits of this regimen. Additionally, all subgroup analyses were *post-hoc* exploratory comparisons without pre-specified stratification criteria, so the reliability of these subgroup findings requires external validation. Prospective RCTs are needed to further confirm the heterogeneous treatment responses across distinct patient subgroups. Second, an important statistical limitation lies in the imbalanced censoring status across the two propensity-matched cohorts. After 1:1 PSM, the TACE+D+C group presented substantially higher censoring rates than the TACE+D group for both OS (35.78% vs 12.84%) and PFS (41.28% vs 7.34%). All censoring events were administrative censoring stemming from the fixed January 2026 data cutoff, with zero patient loss to follow-up throughout surveillance. The higher proportion of event-free patients in the triple therapy cohort at the analysis endpoint may bias the magnitude of observed survival associations. To mitigate this confounding factor, we performed a suite of sensitivity analyses incorporating IPTW and Rosenbaum bounds testing; consistent survival correlation directions and statistical significance were reproduced across all tests, indicating our core survival findings are not exclusively attributable to differential censoring. Notably, both groups had relatively limited median follow-up durations (13.92 months for TACE+D+C, 10.43 months for TACE+D), which introduces two separate analytical constraints. For survival endpoints, the short observational window prevents reliable evaluation of sustained long-term survival patterns. For safety profiling, the truncated follow-up fails to fully capture delayed, low-frequency, and late-emerging AEs, particularly mild chronic AEs associated with targeted and immunotherapeutic agents. Collectively, these limitations highlight the need for future prospective studies with prolonged, standardized long-term safety monitoring. Third, as this was a retrospective analysis, we could not determine the optimal sequential administration timing, dosing intervals, and individualized dose modification strategies for TACE, donafenib, and camrelizumab. Additionally, only all-cause death and tumor progression events were collected to calculate OS and PFS, without standardized prospective documentation of treatment discontinuation, administration delays, and treatment-related deaths. This precluded quantitative assessment of the confounding effects of treatment interruptions or delays on survival outcomes, as well as separate statistical quantification of the proportion of deaths directly attributable to TRAEs. Accordingly, future prospective trials should adopt standardized recording protocols for the aforementioned treatment implementation indicators to enable refined analyses exploring correlations between treatment delivery patterns, safety profiles, and patient prognosis. Fourth, from a translational perspective, this study lacks biomarker-related exploratory analyses. As the combination regimen includes camrelizumab, analysis of molecular or clinical predictive markers (such as PD-L1 expression, peripheral immune cell profiles, and circulating cytokines) would help screen populations most likely to gain therapeutic benefits. However, paired tumor tissue and serial peripheral blood samples were not routinely preserved for biomarker detection during patient treatment and follow-up in our two clinical centers, so we could not perform stratified analyses based on immune-related molecular indicators. Future prospective studies with standardized collection of tissue and liquid biopsy specimens are warranted to identify predictive biomarkers for this triple combination therapy, enabling more precise individualized treatment. Finally, this retrospective cohort did not systematically collect data on immune-related hepatitis, serial laboratory monitoring for HBV reactivation, and standardized antiviral prophylaxis regimens. Given the high proportion of HBV-associated HCC among patients with PVTT in this study, the lack of the above data limits our comprehensive evaluation of liver-specific irAEs and the risk of HBV reactivation during combination immunotherapy. In future research, we will design a prospective follow-up study to document the incidence of immune-related hepatitis, regular HBV virological monitoring results, and complete antiviral treatment regimens.

In conclusion, this PSM retrospective cohort analysis observed an association between receipt of TACE+D+C therapy and longer survival as well as improved disease control, alongside manageable TRAEs, compared with TACE+D monotherapy among patients with HCC and PVTT. *Post-hoc* subgroup analyses detected numerical differences in survival correlations across patient strata; individuals with an ECOG score of 0, tumor size > 7 cm, or PVTT: III-IV displayed a correlative trend of longer survival when receiving triple therapy. These subgroup correlative trends cannot be regarded as definitive therapeutic benefits and warrant external validation in separate cohorts. Importantly, due to the limitations inherent to the retrospective study design, our retrospective observational findings cannot establish causal evidence to confirm the clinical superiority of the triple combination regimen over TACE+D alone. Nevertheless, these data lay a solid foundation for subsequent prospective RCTs to clarify its clinical value.

## Data Availability

The original contributions presented in the study are included in the article/**Supplementary Material**. Further inquiries can be directed to the corresponding author.
