# Supplementary material for: Efficacy and safety of transarterial chemoembolization combined with donafenib and camrelizumab for hepatocellular carcinoma with portal vein tumor thrombus: a dual-center, retrospective propensity score-matched analysis
Source: Front Immunol. 2026 Aug 5;17:1882605. doi: 10.3389/fimmu.2026.1882605 (PMC13485731; doi:10.3389/fimmu.2026.1882605)
Supplement: Supplementary file 1 [file Table1.docx]

Supplementary Materials

Transarterial Chemoembolization (TACE) Procedure

After routine skin disinfection, local infiltration anesthesia was administered at the point of maximal pulsation of the left radial artery. Under ultrasound guidance, vascular puncture was performed via the Seldinger technique, followed by insertion of a 5F vascular sheath. A single-curved catheter was placed into the celiac trunk for arteriography to delineate the feeding arteries, branch distribution, location, and size of the tumor. Subsequently, a 2.7F APT microcatheter was used for super-selective catheterization of the tumor-feeding arteries. Intra-arterial chemotherapy was administered with oxaliplatin 50 mg, epirubicin 30 mg, and fluorouracil 0.75 g. Thereafter, an emulsion composed of ethiodized poppy seed oil injection combined with epirubicin 20 mg was infused for embolization. If necessary, additional embolic agents, including polyvinyl alcohol (PVA) particles (350-560 μm), gelatin sponge particles (560-710 μm), or embolic microspheres (350-560 μm), were supplemented. The embolization endpoint was strictly implemented in accordance with the Clinical Practice Guidelines for TACE Treatment of Hepatocellular Carcinoma in China (1).

Propensity Score Matching (PSM) Analysis

In this study, logistic regression was used to generate propensity scores for 1:1 nearest-neighbour matching with a caliper of 0.2 standard deviations. Matching covariates comprised ECOG performance status, Child-Pugh grade, tumor size, Number of tumors, Portal Vein Tumor Thrombus (PVTT), extrahepatic metastasis, alanine aminotransferase, albumin, and alpha-fetoprotein (AFP). Age and sex were omitted from the primary model following standard confounder selection rules for observational analyses, which require variables to associate with both treatment assignment and survival endpoints to mitigate confounding effectively (2). Baseline comparisons showed negligible intergroup disparities in age and sex (all pre-matching standardized mean difference (SMD) ≤ 0.15), and clinical decisions to administer camrelizumab were guided by liver function, tumor burden, and PVTT grade rather than patient age or sex. Consistent with published PVTT-HCC cohorts and our internal multivariate Cox models, neither variable independently predicted overall survival (OS) or progression-free survival (PFS) after adjustment for core disease-related covariates (3, 4). Including age and sex would therefore inflate model dimensionality without reducing residual confounding. We further validated result stability via supplementary 1:1 PSM and inverse probability of treatment weighting (IPTW) sensitivity analyses incorporating age and sex.

Robustness analysis

To verify the robustness of the PSM results, three robustness analyses and one Rosenbaum sensitivity analysis were performed.

1. 1:2 nearest-neighbor matching algorithm (caliper width set to 0.2 times the standard deviation of propensity score, without replacement)

Propensity scores were constructed using a logistic regression model. Core clinical covariates incorporated in the model consisted of Eastern Cooperative Oncology Group (ECOG), Child-Pugh classification, tumor diameter, tumor number, PVTT, extrahepatic metastasis, alanine aminotransferase (ALT), serum albumin, and AFP.

After PSM, 144 patients were allocated to the TACE+D group and 111 patients to the TACE+D+C group, with partial baseline characteristics remaining imbalanced between the two cohorts. The median overall survival (mOS) was significantly prolonged in the TACE+D+C group versus the TACE+D group (14.20 months, 95% CI: 11.40-20.10 months vs 9.20 months, 95% CI: 6.80-11.40 months; Hazard Ratio (HR) = 0.55, 95% CI: 0.41-0.74, *P* < 0.001). The median progression-free survival (mPFS) was numerically longer in the TACE+D+C group than in the TACE+D group (8.40 months, 95% CI: 6.40-10.00 months vs 6.65 months, 95% CI: 5.30-7.90 months), yet the difference did not reach statistical significance (HR = 0.86, 95% CI: 0.66-1.10, *P* = 0.225).

2. 1:1 nearest-neighbor matching (caliper width set to 0.1 times the standard deviation of propensity score, without replacement)

Propensity scores were established via logistic regression modelling. Key clinical covariates incorporated in the model included ECOG performance status, Child-Pugh grade, tumor diameter, tumor number, PVTT, extrahepatic metastasis, ALT, albumin, and AFP. Sex and age were excluded from the analytical model, as previous investigations have indicated that these variables offer no definitive prognostic predictive value for OS and PFS.

Following PSM, 106 patients were enrolled in both the TACE+D group and the TACE+D+C group. Baseline characteristics were well balanced between the two cohorts, although the retained sample size was relatively limited. mOS was markedly prolonged in the TACE+D+C group relative to the TACE+D group (12.55 months, 95% CI: 9.20-15.40 vs 9.00 months, 95% CI: 6.80-11.00; HR = 0.62, 95% CI: 0.47-0.81, *P* < 0.001). Similarly, mPFS was also significantly longer in the TACE+D+C group than in the TACE+D group (8.90 months, 95% CI: 6.90-11.40 vs 6.75 months, 95% CI: 5.20-8.70), with a statistically significant difference observed (HR = 0.70, 95% CI: 0.53-0.93, *P* = 0.012).

3. 1:1 nearest-neighbor PSM (caliper=0.2 SD) with age+sex included

After PSM, 75 patients were allocated to the TACE+D group and 75 patients to the TACE+D+C group, with partial baseline characteristics remaining imbalanced between the two cohorts. The mOS was significantly prolonged in the TACE+D+C group versus the TACE+D group (12.50 months, 95% CI: 9.80-15.90 months vs 9.00 months, 95% CI: 6.20-11.00 months; HR = 0.55, 95% CI: 0.40-0.78, *P* < 0.001). The mPFS was numerically longer in the TACE+D+C group than in the TACE+D group (8.90 months, 95% CI: 6.90-10.40 months vs 6.80 months, 95% CI: 5.00-8.80 months; HR = 0.697, 95% CI: 0.50-0.97, *P* = 0.030).

4. IPTW analysis(2).

Logistic regression was utilised to calculate propensity scores, with the same covariates incorporated as those applied in the preceding robustness analysis. mOS was significantly longer in the TACE+D+C group than in the TACE+D group (15.4 months, 95% CI: 12.62-20.11 vs 9.2 months, 95% CI: 7.0-11.4; HR = 0.516, 95% CI: 0.39-0.68, *P* < 0.001). Likewise, mPFS was markedly prolonged in the TACE+D+C cohort relative to the TACE+D cohort (11.8 months, 95% CI: 8.91-15.62 vs 7.1 months, 95% CI: 5.52-9.12; HR = 0.510, 95% CI: 0.37-0.69, *P* < 0.001). Consistent findings were obtained across multiple analytical approaches, all of which were consistent with the interpretation that the TACE+D+C regimen may offer improved therapeutic efficacy relative to TACE+D monotherapy, lending additional support to the primary findings.

5. IPTW analysis with age+sex included

Logistic regression was utilised to calculate propensity scores, with the same covariates incorporated as those applied in the preceding robustness analysis. mOS was significantly longer in the TACE+D+C group than in the TACE+D group (15.6 months, 95% CI: 13.12-21.34 vs 9.2 months, 95% CI: 7.00-11.40; HR = 0.51, 95% CI: 0.38-0.67, *P* < 0.001). Likewise, mPFS was markedly prolonged in the TACE+D+C cohort relative to the TACE+D cohort (13.3 months, 95% CI: 8.90-16.40 vs 7.1 months, 95% CI: 5.50-9.12; HR = 0.49, 95% CI: 0.36-0.68, *P* < 0.001).

Rosenbaum analysis(5).

To evaluate the robustness of PSM findings against unmeasured confounding factors, a Rosenbaum sensitivity analysis was performed on the original survival data(6). The upper-bound *P*-values were calculated across a range of gamma values (from 1.0 to 2.0 at an increment of 0.2) (**Table 1**). The results indicated that the observed therapeutic effect lost statistical significance at a gamma value of 1.8, with an upper-bound *P*-value of 0.070. This finding suggested that an unmeasured confounder would need to elevate the odds ratio of treatment assignment by at least 1.8-fold to fully attenuate the significant association between treatment regimen and survival outcomes. Accordingly, our primary results were confirmed to be robust to potential unmeasured confounding.

**Table 1** Upper bound of P-values under different gamma values

| Gamma | ***P***-Lower bound | ***P***-Upper bound |
| --- | --- | --- |
| 1.0 | 0 | 0.0000 |
| 1.2 | 0 | 0.0001 |
| 1.4 | 0 | 0.0023 |
| 1.6 | 0 | 0.0175 |
| 1.8 | 0 | 0.0700 |
| 2.0 | 0 | 0.1807 |

References

1.	[Chinese Clinical Practice Guidelines for Transarterial Chemoembolization of Hepatocellular Carcinoma (2023 Edition)]. *Zhonghua Yi Xue Za Zhi* (2023) 103(34):2674-94. Epub 2023/09/07. doi: 10.3760/cma.j.cn112137-20230630-01114.

2.	Austin PC, Stuart EA. Moving Towards Best Practice When Using Inverse Probability of Treatment Weighting (Iptw) Using the Propensity Score to Estimate Causal Treatment Effects in Observational Studies. *Stat Med* (2015) 34(28):3661-79. Epub 2015/08/05. doi: 10.1002/sim.6607.

3.	Radu IP, Scheiner B, Schropp J, Delgado MG, Schwacha-Eipper B, Jin C, et al. The Influence of Sex and Age on Survival in Patients with Hepatocellular Carcinoma. *Cancers (Basel)* (2024) 16(23). Epub 2024/12/17. doi: 10.3390/cancers16234023.

4.	Li HM, Huang W, Hu C, Zhang ZS, Xiao YD, Wang TC. Prognostic Factors for Patients with Proliferative Hepatocellular Carcinoma after Liver Resection. *J Hepatocell Carcinoma* (2023) 10:2059-71. Epub 2023/11/29. doi: 10.2147/jhc.S440636.

5.	Rosenbaum PR, Rubin DB. The Central Role of the Propensity Score in Observational Studies for Causal Effects. *Biometrika* 70(1):41-55. doi: 10.1093/biomet/70.1.41.

6.	McCandless LC, Gustafson P, Levy AR. A Sensitivity Analysis Using Information About Measured Confounders Yielded Improved Uncertainty Assessments for Unmeasured Confounding. *J Clin Epidemiol* (2008) 61(3):247-55. Epub 2008/01/30. doi: 10.1016/j.jclinepi.2007.05.006.

Events and censoring distribution for OS and PFS after PSM

| Group | Total Cases | OS Death Events | OS Censored Cases | OS Censoring Rate | mOS Censoring Time (Months) | PFS Progression Events | PFS Censored Cases | PFS Censoring Rate | mPFS Censoring Time (Months) |
| --- | --- | --- | --- | --- | --- | --- | --- | --- | --- |
| TACE+D | 109 | 95 | 14 | 12.84% | 10.5 | 101 | 8 | 7.34% | 7.55 |
| TACE+D+C | 109 | 70 | 39 | 35.78% | 15.2 | 64 | 45 | 41.28% | 12.3 |

Abbreviations: OS, overall survival; PFS, progression-free survival; TACE, transarterial chemoembolization; D, donafenib; C, camrelizumab; PSM, propensity score matching; mOS, median overall survival; mPFS, median progression-free survival;

Schoenfeld residual tests

The Schoenfeld residual test was performed to verify the proportional hazards assumption for all Cox regression models. For both OS (table 1) and PFS (table 2) multivariable models, the correlation between Schoenfeld residuals and survival time for all covariates (including treatment group) yielded *P* > 0.05, confirming that the PH assumption was fully satisfied.

Table 1 Schoenfeld residual test for proportional hazards assumption (OS multivariable Cox model)

| Variable | Correlation coefficient | PH assumption satisfied | *P*-value |
| --- | --- | --- | --- |
| Treatment group (TACE+D+C vs TACE+D) | 0.082 | *P* > 0.05 | 0.415 |
| Age (years): ≤ 40; > 40 | 0.057 | *P* > 0.05 | 0.582 |
| Sex: Female; Male | 0.061 | *P* > 0.05 | 0.554 |
| HBV: No; Yes | 0.049 | *P* > 0.05 | 0.631 |
| ECOG score: 0; 1 | 0.073 | *P* > 0.05 | 0.472 |
| Child-Pugh class: A; B | 0.091 | *P* > 0.05 | 0.368 |
| AFP (ng/mL): ≤ 400; > 400 | 0.068 | *P* > 0.05 | 0.501 |
| ALT (U/L): ≤ 35; > 35 | 0.052 | *P* > 0.05 | 0.608 |
| Albumin (g/L): ≤ 30; > 30 | 0.088 | *P* > 0.05 | 0.385 |
| Extrahepatic metastasis: Yes; No | 0.079 | *P* > 0.05 | 0.432 |
| Number of tumors: ≤ 3; > 3 | 0.045 | *P* > 0.05 | 0.663 |
| Tumor size: ≤ 7 cm; > 7 cm | 0.095 | *P* > 0.05 | 0.347 |
| PVTT: I-II; III-IV | 0.064 | *P* > 0.05 | 0.527 |

Table 2 Schoenfeld residual test for proportional hazards assumption (PFS multivariable Cox model)

| Variable | Correlation coefficient | PH assumption satisfied | *P*-value |
| --- | --- | --- | --- |
| Treatment group (TACE+D+C vs TACE+D) | 0.076 | *P* > 0.05 | 0.453 |
| Age (years): ≤ 40; > 40 | 0.051 | *P* > 0.05 | 0.615 |
| Sex: Female; Male | 0.067 | *P* > 0.05 | 0.512 |
| HBV: No; Yes | 0.044 | *P* > 0.05 | 0.672 |
| ECOG score: 0; 1 | 0.071 | *P* > 0.05 | 0.489 |
| Child-Pugh class: A; B | 0.089 | *P* > 0.05 | 0.376 |
| AFP (ng/mL): ≤ 400; > 400 | 0.065 | *P* > 0.05 | 0.538 |
| ALT (U/L): ≤ 35; > 35 | 0.050 | *P* > 0.05 | 0.621 |
| Albumin (g/L): ≤ 30; > 30 | 0.085 | *P* > 0.05 | 0.401 |
| Extrahepatic metastasis: Yes; No | 0.077 | *P* > 0.05 | 0.445 |
| Number of tumors: ≤ 3; > 3 | 0.042 | *P* > 0.05 | 0.689 |
| Tumor size: ≤ 7 cm; > 7 cm | 0.092 | *P* > 0.05 | 0.359 |
| PVTT: I-II; III-IV | 0.063 | *P* > 0.05 | 0.541 |

Abbreviation: HBV, Hepatitis B Virus; AFP, Alpha-Fetoprotein; ALT, Alanine Aminotransferase; ECOG, Eastern Cooperative Oncology Group; PVTT, Portal Vein Tumor Thrombus; TACE+D, Transarterial Chemoembolization combined with donafenib; TACE+D+C, Transarterial Chemoembolization combined with donafenib and camrelizumab; PH, proportional hazards;

**Supplementary Figure 1** Forest plot of subgroup analysis for OS between the TACE+D group and the TACE+D+C group.

Abbreviation: HBV, Hepatitis B Virus; AFP, Alpha-Fetoprotein; ALT, Alanine Aminotransferase; ECOG, Eastern Cooperative Oncology Group; PVTT, Portal Vein Tumor Thrombus; CI, Confidence Interval;

**Supplementary Figure 2** Kaplan-Meier survival curves for OS and PFS in the stratified analysis.

Abbreviation: ECOG, Eastern Cooperative Oncology Group; PVTT, Portal Vein Tumor Thrombus; TACE+D, Transarterial Chemoembolization combined with donafenib; TACE+D+C, Transarterial Chemoembolization combined with donafenib and camrelizumab; 95% CI, 95% Confidence Interval;

**Supplementary Table 1** Tumor response evaluation of intrahepatic nodules in the two groups before and after PSM.

| Tumor response, n (%) | Before PSM | | | After PSM | | |
| --- | --- | --- | --- | --- | --- | --- |
|  | TACE+D group(n=152) | TACE+D+C group(n=133) | *P-*value | TACE+D group(n=109) | TACE+D+C group(n=109) | *P-*value |
| CR | 8 (5.26) | 17 (12.78) | 0.025 | 4 (3.67) | 13 (11.93) | 0.023 |
| PR | 42 (27.63) | 53 (39.85) | 0.029 | 36 (33.03) | 51 (46.79) | 0.038 |
| SD | 68 (44.74) | 45 (33.83) | 0.061 | 44 (40.37) | 34 (31.19) | 0.158 |
| PD | 34 (22.37) | 18 (13.53) | 0.054 | 25 (22.94) | 11 (10.09) | 0.011 |
| ORR | 50 (32.89) | 70 (52.63) | <0.001 | 40 (36.70) | 64 (58.72) | 0.001 |
| DCR | 118 (77.63) | 115 (86.47) | 0.054 | 84 (77.06) | 98 (89.91) | 0.011 |

Abbreviation: PSM, Propensity Score Matching; CR, Complete Response; PR, Partial Response; SD, Stable Disease; PD, Progressive Disease; ORR, Objective Response Rate; DCR, Disease Control Rate; TACE+D, Transarterial Chemoembolization combined with donafenib; TACE+D+C, Transarterial Chemoembolization combined with donafenib and camrelizumab;

**Supplementary Table 2** Tumor response evaluation of portal vein tumor thrombus in the two groups before and after PSM.

| Tumor response, n (%) | Before PSM | | | After PSM | | |
| --- | --- | --- | --- | --- | --- | --- |
|  | TACE+D group (n = 152) | TACE+D+C group (n = 133) | *P-*value | TACE+D group (n = 109) | TACE+D+C group (n = 109) | *P-*value |
| CR | 5 (3.29) | 10 (7.52) | 0.111 | 2 (1.83) | 6 (5.50) | 0.280 |
| PR | 30 (19.74) | 45 (33.83) | 0.007 | 23 (21.10) | 40 (36.70) | 0.011 |
| SD | 59 (38.82) | 41 (30.83) | 0.159 | 45 (41.28) | 38 (34.86) | 0.329 |
| PD | 58 (38.16) | 37 (27.82) | 0.065 | 39 (35.78) | 25 (22.94) | 0.037 |
| ORR | 35 (23.03) | 55 (41.35) | <0.001 | 26 (23.85) | 46 (42.20) | 0.004 |
| DCR | 94 (61.84) | 96 (72.18) | 0.065 | 70 (64.22) | 84 (77.06) | 0.037 |

Abbreviation: PSM, Propensity Score Matching; CR, Complete Response; PR, Partial Response; SD, Stable Disease; PD, Progressive Disease; ORR, Objective Response Rate; DCR, Disease Control Rate; TACE+D, Transarterial Chemoembolization combined with donafenib; TACE+D+C, Transarterial Chemoembolization combined with donafenib and camrelizumab;

**Supplementary Figure 3** AEs associated with the TACE+D+C and TACE+D groups

Abbreviation: TACE+D, Transarterial Chemoembolization combined with donafenib; TACE+D+C, Transarterial Chemoembolization combined with donafenib and camrelizumab; RCCEP, Reactive Cutaneous Capillary Endothelial Proliferation; AEs, Adverse Events; TRAEs, Treatment-Related Adverse Events; ALT, Alanine Aminotransferase;

Supplementary Table 3 TRAEs between the two groups

| AEs, n (%) | **Grade 1-2 TRAEs** | | | **Grade 3-4 TRAEs** | | |
| --- | --- | --- | --- | --- | --- | --- |
|  | TACE+D group (n = 109) | TACE+D+C group (n = 109) | *P-*value | TACE+D group (n = 109) | TACE+D+C group (n = 109) | *P-*value |
| **TRAEs** |  |  |  |  |  |  |
| Diarrhea | 17(15.61) | 16(14.68) | 0.850 | 5(4.59) | 3(2.75) | 0.719 |
| Hair loss | 24(22.02) | 26(23.85) | 0.747 | 0 | 0 | 0 |
| Rash | 8(7.34) | 4(3.67) | 0.235 | 0 | 0 | 0 |
| Hand-foot skin reaction | 27(24.77) | 28(25.69) | 0.876 | 8(7.34) | 9(8.26) | 0.801 |
| **irAEs** |  |  |  |  |  |  |
| Hypothyroidism | 0(0) | 5(4.59) | 0.070 | 0 | 0 | 0 |
| RCCEP | 0(0) | 13(11.93) | <0.001 | 0 | 0 | 0 |
| IRP | 0(0) | 9(8.26) | 0.006 | 0 | 0 | 0 |
| **AEs after TACE** |  |  |  |  |  |  |
| Abdominal pain | 62(56.88) | 66(60.55) | 0.582 | 5(4.59) | 6(5.5) | 0.517 |
| Fever | 20(18.35) | 16(14.68) | 0.466 | 0 | 0 | 0 |
| Vomiting | 31(28.44) | 35(32.11) | 0.555 | 4(3.67) | 2(1.83) | 0.679 |
| Increased ALT | 41(37.61) | 30(27.52) | 0.112 | 0 | 0 | 0 |

Abbreviations: RCCEP, reactive cutaneous capillary endothelial proliferation; TACE+D, Transarterial Chemoembolization combined with donafenib; TACE+D+C, Transarterial Chemoembolization combined with donafenib and camrelizumab; irAEs, immune-related adverse events; TRAEs, Treatment-Related Adverse Events; AEs, Adverse Events; IRP, Immune-related pneumonitis;

Supplementary Table 4 Univariate and multivariate cluster-robust Cox regression analyses for OS after PSM

| Variable | Univariate analysis | | | | | Multivariate analysis | | | | |
| --- | --- | --- | --- | --- | --- | --- | --- | --- | --- | --- |
|  | HR | Conventional SE | Robust SE | 95% CI | *P*-value | HR | Conventional SE | Robust SE | 95% CI | *P*-value |
| Treatment group (TACE+D+C vs TACE+D) | 0.38 | 0.16 | 0.15 | 0.28–0.51 | <0.001 | 0.29 | 0.16 | 0.17 | 0.21–0.41 | <0.001 |
| Age (years): ≤ 40; > 40 | 0.96 | 0.25 | 0.22 | 0.61–1.51 | 0.862 |  |  |  |  |  |
| Sex: Female; Male | 0.82 | 0.19 | 0.20 | 0.55–1.22 | 0.329 |  |  |  |  |  |
| HBV: No; Yes | 1.18 | 0.17 | 0.17 | 0.83–1.67 | 0.353 |  |  |  |  |  |
| ECOG score: 0; 1 | 2.36 | 0.21 | 0.23 | 1.48–3.76 | <0.001 | 1.67 | 0.22 | 0.23 | 1.07–2.63 | 0.025 |
| Child-Pugh class: A; B | 3.71 | 0.17 | 0.16 | 2.67–5.16 | <0.001 |  |  |  |  |  |
| AFP (ng/mL): ≤ 400; > 400 | 1.37 | 0.16 | 0.16 | 1.00–1.89 | 0.051 |  |  |  |  |  |
| ALT (U/L): ≤ 35; > 35 | 1.59 | 0.17 | 0.16 | 1.16–2.20 | 0.004 |  |  |  |  |  |
| Albumin (g/L): ≤ 30; > 30 | 0.21 | 0.17 | 0.17 | 0.15–0.29 | <0.001 | 0.87 | 0.02 | 0.02 | 0.84–0.91 | <0.001 |
| Extrahepatic metastasis: Yes; No | 1.84 | 0.18 | 0.22 | 1.19–2.83 | 0.006 | 1.59 | 0.19 | 0.20 | 1.06–2.40 | 0.026 |
| Number of tumors: ≤ 3; > 3 | 1.04 | 0.18 | 0.18 | 0.72–1.49 | 0.833 |  |  |  |  |  |
| Tumor size: ≤ 7 cm; > 7 cm | 0.97 | 0.17 | 0.16 | 0.69–1.35 | 0.867 |  |  |  |  |  |
| PVTT: I-II; III-IV | 2.03 | 0.16 | 0.15 | 1.49–2.77 | <0.001 | 2.09 | 0.17 | 0.19 | 1.43–3.05 | <0.001 |

Notes: All Cox models adopted cluster-adjusted robust sandwich standard errors clustered by PSM matching pairs to eliminate within-pair correlation of matched patients. Conventional model-based standard errors without cluster correction are listed for comparative reference. Covariates with univariate two-sided *P* < 0.1, together with the mandatory treatment group, were entered into bidirectional stepwise multivariate models. *P*-values less than 0.001 are presented as < 0.001;

Note: Reference groups for each categorical variable are defined as follows: ECOG score = 0; albumin ≤30 g/L; no extrahepatic metastasis; PVTT I-II; treatment group = TACE+D; Child-Pugh class A; AFP ≤ 400 ng/ml; ALT ≤ 35 U/L; age ≤ 40 years; number of tumors ≤ 3; tumor size ≤ 7 cm; sex = female. HR > 1 indicates a higher risk of endpoint events in the comparison group relative to the reference group; HR < 1 indicates a lower risk.

Abbreviations: HR, hazard ratio; SE, standard error; CI, confidence interval; PSM, propensity score matching; TACE, transarterial chemoembolization; D, donafenib; C, camrelizumab; ECOG, Eastern Cooperative Oncology Group; HBV, hepatitis B virus; AFP, alpha-fetoprotein; ALT, alanine transaminase; PVTT, portal vein tumor thrombus.

Supplementary Table 5 Univariate and multivariate cluster-robust Cox regression analyses for PFS after PSM

| Variable | Univariate analysis | | | | | Multivariate stepwise analysis | | | | |
| --- | --- | --- | --- | --- | --- | --- | --- | --- | --- | --- |
|  | HR | Conventional SE | Robust SE | 95% CI | *P*-value | HR | Conventional SE | Robust SE | 95% CI | *P*-value |
| Treatment group (TACE+D+C vs TACE+D) | 0.35 | 0.164 | 0.160 | 0.26–0.48 | <0.001 | 0.29 | 0.169 | 0.168 | 0.21–0.41 | <0.001 |
| Age (years): ≤ 40; > 40 | 0.94 | 0.257 | 0.242 | 0.59–1.51 | 0.796 |  |  |  |  |  |
| Sex: Female; Male | 0.77 | 0.192 | 0.187 | 0.54–1.12 | 0.172 |  |  |  |  |  |
| HBV: No; Yes | 1.07 | 0.177 | 0.176 | 0.76–1.51 | 0.712 |  |  |  |  |  |
| ECOG score: 0; 1 | 2.19 | 0.218 | 0.208 | 1.45–3.29 | <0.001 | 1.66 | 0.222 | 0.217 | 1.09–2.55 | 0.019 |
| Child-Pugh class: A; B | 3.51 | 0.172 | 0.179 | 2.47–4.99 | <0.001 |  |  |  |  |  |
| AFP (ng/mL): ≤ 400; > 400 | 1.40 | 0.165 | 0.165 | 1.01–1.93 | 0.042 |  |  |  |  |  |
| ALT (U/L): ≤ 35; > 35 | 1.56 | 0.175 | 0.165 | 1.13–2.15 | 0.007 |  |  |  |  |  |
| Albumin (g/L): ≤ 30; > 30 | 0.25 | 0.172 | 0.195 | 0.17–0.37 | <0.001 | 0.88 | 0.021 | 0.021 | 0.84–0.91 | <0.001 |
| Extrahepatic metastasis: Yes; No | 1.66 | 0.185 | 0.217 | 1.09–2.54 | 0.019 | 1.44 | 0.195 | 0.197 | 0.97–2.12 | 0.064 |
| Number of tumors: ≤ 3; > 3 | 1.14 | 0.185 | 0.179 | 0.80–1.62 | 0.461 |  |  |  |  |  |
| Tumor size: ≤ 7 cm; > 7 cm | 0.99 | 0.174 | 0.166 | 0.71–1.37 | 0.945 |  |  |  |  |  |
| PVTT: I-II; III-IV | 1.86 | 0.162 | 0.162 | 1.36–2.56 | <0.001 | 1.78 | 0.175 | 0.199 | 1.20–2.63 | 0.002 |

Notes: All Cox models adopted cluster-adjusted robust sandwich standard errors clustered by PSM matching pairs to eliminate within-pair correlation of matched patients. Conventional model-based standard errors without cluster correction are listed for comparative reference. Covariates with univariate two-sided *P* < 0.1, together with the mandatory treatment group, were entered into bidirectional stepwise multivariate models. *P*-values less than 0.001 are presented as < 0.001;

Note: Reference groups for each categorical variable are defined as follows: ECOG score = 0; albumin ≤30 g/L; no extrahepatic metastasis; PVTT I-II; treatment group = TACE+D; Child-Pugh class A; AFP ≤ 400 ng/ml; ALT ≤ 35 U/L; age ≤ 40 years; number of tumors ≤ 3; tumor size ≤ 7 cm; sex = female. HR > 1 indicates a higher risk of endpoint events in the comparison group relative to the reference group; HR < 1 indicates a lower risk.

Abbreviations: HR, hazard ratio; SE, standard error; CI, confidence interval; PSM, propensity score matching; TACE, transarterial chemoembolization; D, donafenib; C, camrelizumab; ECOG, Eastern Cooperative Oncology Group; HBV, hepatitis B virus; AFP, alpha-fetoprotein; ALT, alanine transaminase; PVTT, portal vein tumor thrombus.
